# Some Remarks on the Treatment of Uncomplicated Wounds and Ulcers

**Published:** 1889-04

**Authors:** B. E. Hadra

**Affiliations:** Professor of Surgery, Texas Medical College, Galveston


### SOME REMARKS ON THE TREATMENT OF UNCOMPLICATED WOUNDS AND ULCERS.

BY B. E. HADRA, M. D.,

*Professor of Surgery, Texas Medical College, Galveston.*

[Read before Galveston Medical Club, and furnished to DANIEL'S TEXAS MEDICAL JOURNAL for publication.]

ACCORDING to your rules, it is my duty to read a paper before you to-night as an introductory to your club. I was told that the shorter it would be, the more it would be appreciated. I hope then that in this respect you will be gratified.

Simplicity is a virtue, perhaps nowhere more so than in surgery. The simpler therefore the treatment of a large class of surgical ailments, the better able will we be to accomodate the patient and ourselves.

According to the present views on inflammation and ulcerations, it is the pathogenic germ which causes the abnormal changes, sufficient to prevent the injured tissues from repairing themselves by the natural process of healing. We may take it for granted that any break of sound tissue would, if left alone, and if kept in apposition, be remedied by a reparative process, endowed to it by nature. If this be true, then the best thing for us to do would be, not to meddle with wounds at all, and in reality this principle is the basis of modern surgery. Uncomplicated wounds which do not require special attention to blood vessels, nerves or tendons, or which are not penetrating into cavities or secretory organs, should simply be closed so as to prevent the pathogenic germ from entering. This is certainly nothing new to you, but I wish to make a point that the methods mostly used to day, are yet doing too much by overdoing asepsis and antisepsis. For cleasning wounds there are used by most of us antiseptic fluids strong enough to kill the germs. But evidently the tissues must also suffer from such foreign substances. The matter stands thus: either the antiseptic solution is not strong enough to kill the germ, or it is, and then not only the pathological but also the normal bodies, with which it comes in contact, will be injured. Of course, where contamination has already occurred, the consideration of destroying the virus, is so preponderating that the care for repair must be secondary. Then strong antiseptis, drainage, etc., is called for;—but where we have good reason to consider a wound surgically clean, be it either after an operation or an accident, then it seems only reasonable not to lessen the chances of first intention by antiseptics. Uncontaminated blood should be simply washed away with pure (boiled) water, using as little rubbing with sponge, etc., as possible, because the minute clots which close the mouths of the vessels will evidently be removed by such harsh treatment, and hemorrhage as well as the possibility of the entrance of virulent material will be increased. Syringing or expressing the aseptic sponge at some height, so as to wash over the wound, should be the rule. The surrounding skin may be well disinfected.

If a wound is once well united, then the suture ought to protect it against the entrance of any foreign substances, and I do not see much necessity then for a full Lister's application, which may irritate surrounding skin. Of course, it is different when drainage is practical. In the first place a good cotton padding and before all a well overlapping piece of (gutta percha) rubber tissue is all that is needed. This latter material deserves a far greater appreciation than it has found among general practioners. Its easily rendered aseptic by being washed in sublimated or carbolized water; it is impermeable, cheap, etc., but before all it is smooth and not irritating; however, to relieve the mind of the patient and surgeon, the sutured part may be dusted with iodoform, bismuth or some similar powder, although the first mentioned one sometimes causes irritation, even to the extent of an eczematous eruption.

If the wound cannot be closed up from one or the other reason, but if it should from all evidence be aseptic, I deem every foreign body, whatever its name may be, whether medicated or not, whether powder or ointment, to be injurious. If strong, it will destroy tissue cells; if mild, it will at least irritate, and under all circumstances, it must as all foreign bodies do, interfere with the natural process of reparation. In my opinion and from my experience, the best then to do, is to clean antiseptically only the surrounding parts, to use nothing but boiled water in the above described manner for the removal of blood clots, etc., and then to cover the wound with a sufficiently large, well disinfected piece of gutta percha or rubber tissue, which after being bathed in antiseptic fluid should be well washed off in simple boiled water so as not to carry any irritating material. Then on top of the tissue a cotton padding may be fastened with a rubber bandage for protection.

The same principles should govern us in the treatment of ulcers, which finally, are nothing but contaminated breaks of the skin with such changes as are produced by the inroad of pathogenic germs into the deeper situated tissues.

Fistulous or specific ulcers I do not intend to consider here. I am confining myself to simple flat ulcers, of which the chronic ulcers of the leg is the best known representative.

Here of course we must use sufficiently strong antiseptics, first, to render the ulcer aseptic. I am very successful with a permanent application of corrosive sublimate solution 1-3000 for this purpose. A wetted cotton pad which is constantly kept moist is applied from 3 to 5 days, wrapped over with a piece of rubber tissue, and a rubber bandage, in order to keep the compress well pressed against the ulcer, as soon as every trace of pus has disappeared nothing should be done but to apply gutta percha tissue directly to sore, and a gauze compress fastened over it. This dressing is not removed for several days, and then only to watch the progress and to satisfy ourselves of a perfect asepsis.

Should pus appear again the whole process is repeated. The patient should not be confined to bed unless venous stasis necessitates rest and an elevation of the affected limb. In this simple way I have seen even the much dreaded callous ulcer of the leg heal quickly and without interfering much with the patient's avocation.

I need not say that where the defect of the skin is very extensive, skin grafting or skin transplantation, may become necessary; but it is surprising how under the above described management even very large ulcers will heal, the borders showing from day to day an extension of the epithelial layer.

Should, however, the assistance of new skin centers be required in the middle of the ulcer, then the treatment need not differ after the transplantation from the one mapped out, and furthermore the transparency of the gutta percha tissue will permit you to watch the progress of the grafts so that you can clean the wounds from secretions whenever they should unexpectedly interfere with attachment of new tissues.

